# Pathogenesis and Therapeutic Advances in Heart Failure with Preserved Ejection Fraction

**DOI:** 10.31083/RCM45613

**Published:** 2026-01-16

**Authors:** Xin Fan, Jie Ma, Xu Zhao, Jing Yu

**Affiliations:** ^1^Department of Cardiology, Lanzhou University Second Hospital, 730030 Lanzhou, Gansu, China; ^2^The Second Clinical Medical College, Lanzhou University, 730030 Lanzhou, Gansu, China

**Keywords:** HFpEF, inflammation, oxidative stress, energy metabolism, therapy

## Abstract

Heart failure with preserved ejection fraction (HFpEF) has progressively emerged as the predominant form of heart failure. Thus, studies on the underlying mechanisms of HFpEF have shifted from pathophysiological to molecular factors. Meanwhile, previous studies have primarily focused on inflammation, oxidative stress, metabolic dysregulation, and impaired cardiac compliance (manifesting as ventricular hypertrophy and interstitial fibrosis). In addition to conventional guideline-directed medical therapies, novel therapeutic strategies targeting these aforementioned pathogenic pathways have been investigated. This review aimed to summarize recent progress in HFpEF pathogenesis and emerging treatment approaches, offering insights for developing novel diagnostic and management strategies.

## 1. Introduction

Heart failure with preserved ejection fraction (HFpEF) is a cardiovascular syndrome characterized primarily by left ventricular diastolic dysfunction. As the aging population and the prevalence of metabolic diseases such as hypertension and obesity increase, the incidence of HFpEF continues to increase. HFpEF has surpassed heart failure with reduced ejection fraction (HFrEF) as the predominant form of heart failure. Studies have indicated that HFpEF constitutes approximately 50% of all heart failure cases [1]. Although its age-specific incidence rate shows a declining trend, the magnitude of this decline is significantly lower than that observed in the case of HFrEF [2].

Studies on the pathological mechanisms of HFpEF have mainly focused on macroscopic hemodynamic characteristics, such as left ventricular diastolic dysfunction, left atrial dysfunction, and epicardial factors. However, these studies have not elucidated its pathogenesis at a deeper and fundamental level. In 2013, Paulus and Tschöpe [3] proposed the inflammation hypothesis to link HFpEF with systemic comorbidities (e.g., obesity, diabetes, and hypertension), suggesting that chronic low-grade inflammation and oxidative stress driven by these comorbidities are key molecular mechanisms leading to myocardial cell dysfunction, coronary microvascular dysfunction (CMD), and myocardial interstitial fibrosis. These findings shifted the focus of research from organ-level pathophysiological factors to cellular and molecular factors, substantially improved the understanding of the pathogenesis of HFpEF, and guided the development of novel therapeutic strategies targeting inflammatory and oxidative stress–related pathways.

Despite preserved systolic function, problems such as decreased exercise tolerance, reduced quality of life, and high hospitalization rates are prevalent among patients with HFpEF, and effective treatments remain lacking. Consequently, elucidating the pathological mechanisms of HFpEF and optimizing therapeutic strategies have become major research focuses in recent years. This review aimed to summarize the pathological mechanisms of HFpEF and discuss recent therapeutic advances, providing insights for improving the diagnosis and treatment of HFpEF.

## 2. Major Mechanisms Underlying Cardiac Diastolic Dysfunction in HFpEF

### 2.1 Inflammation, Oxidative Stress, and Energy Metabolism Synergistically Promote HFpEF Development 

**Fig. 1.**
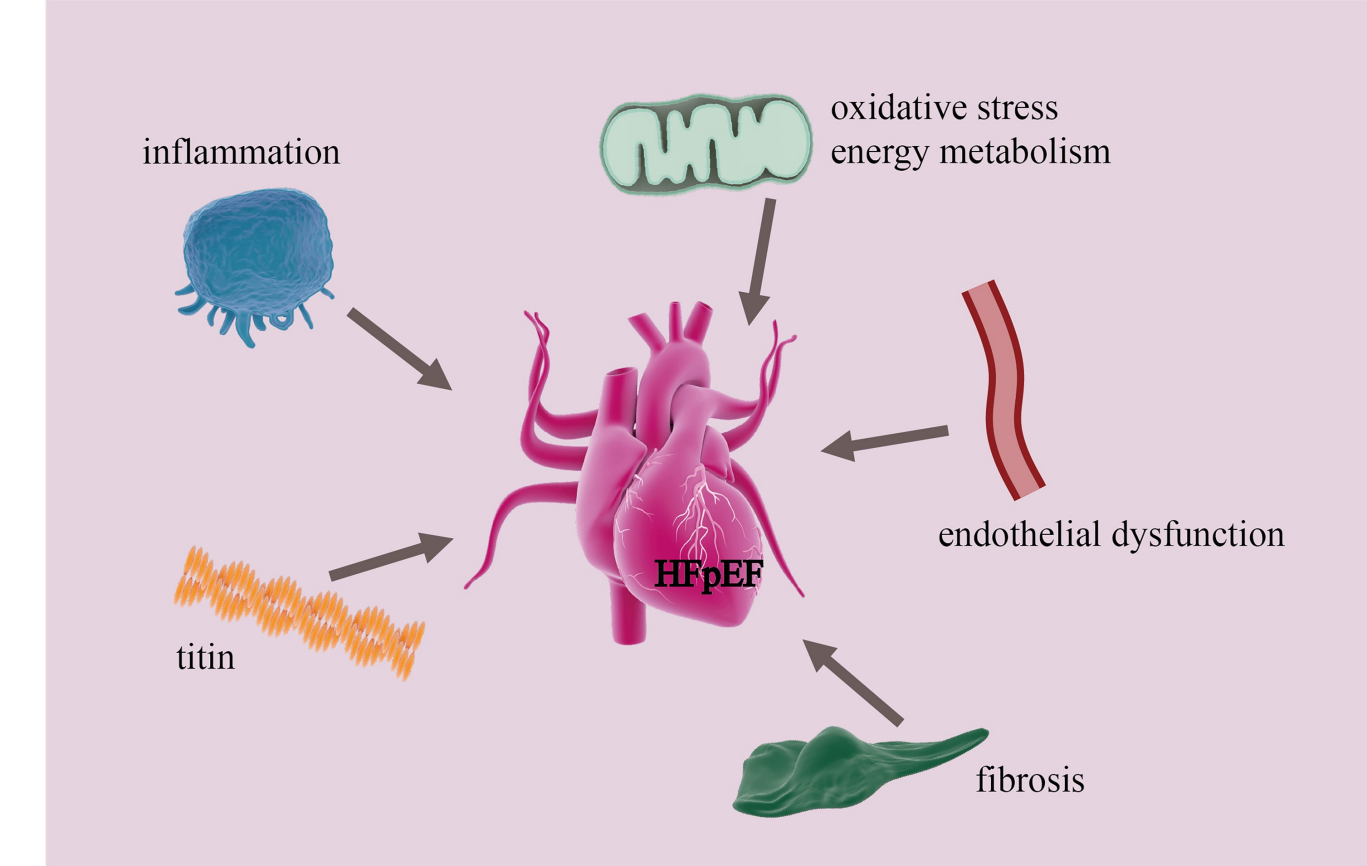
**Major mechanisms underlying cardiac diastolic dysfunction in HFpEF**. HFpEF, Heart failure with preserved ejection fraction.

Analysis of different types of heart failure using SOMAscan technology has revealed that HFpEF exhibits a unique proteomic signature characterized by the upregulation of inflammation-related proteins, such as interleukin-6 receptor fraction (IL-6R), indicating that inflammation is the main factor driving HFpEF pathogenesis (Fig. 1) [4]. Among various inflammatory mediators, the interleukin-6 (IL-6)/IL-6R signaling pathway plays a key role in the development of HFpEF. It not only acts as the main regulator of acute-phase responses but also directly promotes the production of C-reactive protein (CRP) in the liver, amplifying the systemic inflammatory response, and induces cardiac hypertrophy and fibrosis through the gp130–JAK–STAT pathway [5]. Therefore, it is considered the key driver of the inflammatory cascade in HFpEF. Tumor necrosis factor-alpha (TNF-α), another important inflammatory mediator, leads to insulin resistance, apoptosis, and negative inotropic effects in cardiomyocytes by activating the NF-κB pathway. In addition, it is one of the most potent cytokines to activate cardiac fibroblasts, stimulate collagen synthesis, and promote interstitial fibrosis [6]. The formation of neutrophil extracellular traps (NETs) indicates the excessive activation of innate immunity. NETs contribute to the toxicity and dysfunction of coronary microvascular endothelial cells directly through the histones and proteases present in them, such as myeloperoxidase (MPO), and act as antigens to continuously induce immune responses [7]. They serve as an amplifier and effector in the acute exacerbation of disease or specific subtypes (e.g., combined autoimmune diseases). These inflammatory mediators do not work independently but are part of a synergistic network: the release of IL-6 and TNF-α from adipose tissue induced by comorbidities such as obesity and diabetes initiates a systemic low-grade inflammatory state [8]. This state promotes neutrophil activation and NET formation [9, 10]. NET components further damage the microvascular endothelium, releasing more IL-6 and TNF-α and consequently creating a self-reinforcing vicious cycle that results in cardiac injury through pathways such as activation of fibroblasts, stimulation of collagen synthesis, and induction of coronary microvascular endothelial dysfunction.

Oxidative stress is a key mechanism underlying myocardial injury in HFpEF, forming a vicious cycle with inflammation (Fig. 1). Superoxide and hydrogen peroxide (H_2_O_2_) generated from the activation of NADPH oxidase 1 (NOX1) disrupt calcium (Ca^2+^) homeostasis through direct post-translational modifications. On the one hand, reactive oxygen species (ROS) oxidize the key thiol group of the ryanodine receptor type 2 (RyR2) protein and activate Ca^2+^/calmodulin-dependent protein kinase II (CaMKII), resulting in the “hyperopen” state of RyR2 channels. This state leads to Ca^2+^ leakage and depletion in the sarcoplasmic reticulum during relaxation. On the other hand, ROS directly inhibits sarco/endoplasmic reticulum Ca^2+^ ATPase 2a (SERCA2a) pump activity through nitration or glutathione modification, and Ca^2+^ depletion caused by RyR2 leakage suppresses SERCA2a activity, increasing the consumption of adenosine triphosphate (ATP) and exacerbating Ca^2+^ recycling disorders [11, 12, 13, 14]. However, the contribution of diastolic Ca^2+^ dysregulation to injury in HFpEF varies by underlying etiological factors. Ca^2+^ dysregulation is specifically observed in diabetic HFpEF but not in ischemic or hypertensive HFpEF [15].

Abnormal energy metabolism is common among patients with HFpEF (Fig. 1). Compared with age-matched healthy individuals, elderly patients with HFpEF have a 46% lower skeletal muscle mitochondrial content and a 54% lower expression level of the mitochondrial fusion protein mitofusin 2 (MFN2). These alterations are significantly correlated with reduced peak oxygen consumption (peak VO_2_) and 6-minute walk distance, indicating that abnormalities in mitochondrial dynamics are a key contributor to exercise intolerance in HFpEF [16]. It is noteworthy that the extent and underlying mechanisms of these abnormalities vary by the phenotype of HFpEF and are strongly associated with specific clusters of comorbidities. Patients with HFpEF with diabetes mellitus can simultaneously exhibit hyperglycemia-induced accumulation of advanced glycation end products (AGEs). These factors aggravate oxidative stress and directly damage mitochondrial DNA and electron transport chain protein complexes through their receptor (RAGE), leading to more severe impairment of ATP production [17]. Therefore, the reduction of MFN2 expression and mitochondrial content is not the result of a single mechanism but the terminal manifestation of different pathogenic pathways (e.g., lipotoxicity, inflammation, glucotoxicity, and oxidative stress). Understanding their association with specific phenotypes is crucial for the development of precise metabolic therapeutic strategies for HFpEF.

Mitochondrial dynamic imbalance driven by inflammation and oxidative stress, especially excessive fission mediated by dynamin-related protein 1 (Drp1), leads to fragmentation of the mitochondrial network. The disordered formation of cristae leads to abnormal assembly of electron transport chain complexes, which significantly suppresses ATP synthesis and causes energy starvation in cardiomyocytes. Furthermore, fragmented mitochondria increase ROS production owing to inefficient electron transport, whereas the accumulation of dysfunctional mitochondrial fragments impairs energy metabolism, forming a vicious cycle [18, 19]. Inhibiting DRP1 or enhancing the expression of mitochondrial fusion proteins (e.g., MFN2) can improve myocardial energy metabolism and diastolic function [20].

Metabolomic analyses of endomyocardial biopsies from patients with HFpEF have revealed significantly decreased levels of glycolytic intermediates, such as glucose-6-phosphate and fructose-1,6-bisphosphate. Exacerbated myocardial oxidative stress is correlated with the activation of inducible nitric oxide synthase (iNOS). iNOS activation inhibits the Akt signaling pathway through S-nitrosylation, leading to insulin resistance and mitochondrial dysfunction. Concurrently, downregulation of mitochondrial pyruvate carrier protein 1 (MPC1) causes pyruvate accumulation, indicating impaired cardiac glucose metabolism in HFpEF [21, 22]. In addition, angiotensin II (Ang II) and norepinephrine reduce glucose oxidation, which is associated with myocardial hypertrophy and diastolic dysfunction. Targeting these pathways is a novel therapeutic approach for HFpEF [23].

### 2.2 Endothelial Dysfunction as a Pathogenic Mechanism in HFpEF

The prevalence of endothelial dysfunction is higher in patients with HFpEF than in patients with hypertension and healthy individuals [24]. Inflammatory abnormalities triggered by obesity and diabetes impair vascular endothelial soluble guanylate cyclase (sGC), cyclic guanosine monophosphate (cGMP), and protein kinase G (PKG) signaling. This impairment causes endothelial vascular damage, resulting in CMD that diminishes cardiomyocyte protection [25]. Concurrently, increased NOX activity and uncoupled endothelial nitric oxide synthase (eNOS) elevate myocardial superoxide production, further reducing nitric oxide (NO) bioavailability and impairing endothelium-dependent vasodilation in coronary arterioles [26]. Although the prevalence of CMD is similar between male and female patients with HFpEF, its driving factors differ by sex. For instance, inflammatory CMD phenotypes appear predominantly in men, whereas ventricular remodeling and fibrosis are more common in women [27]. This difference can be attributed to the anti-inflammatory properties of estrogen. In endothelial cells, estrogen activates eNOS by rapid signaling through the Phosphoinositide 3-Kinase/Protein Kinase B (PI3K/Akt) pathway to release NO for vasodilation [28]. Therefore, clinicians should pay attention to inflammation levels in male patients with HFpEF and cardiac structure, especially fibrosis, in female patients, and choose treatment plans according to the observed pathological changes (Fig. 1).

### 2.3 Decreased Cardiac Compliance Directly Causes Diastolic Dysfunction in HFpEF 

Increased myocardial stiffness is a primary factor leading to diastolic dysfunction (Fig. 1). It is closely associated with extracellular matrix (ECM) fibrosis and abnormalities in the sarcomeric protein titin.

Titin serves as the primary structural determinant of cardiomyocyte stiffness. The two major cardiac titin isoforms are the more flexible N2BA and the stiffer N2B. A negative N2BA/N2B ratio (i.e., increased N2B expression) is consistently observed in both animal models of HFpEF and cardiac biopsies from patients with HFpEF [29]. HFpEF models also exhibit abnormal titin phosphorylation patterns. Hypophosphorylation occurs at I-band phosphoserine residues (Ser3991, Ser4043, and Ser4080) and Ser12884 in the PEVK domain, whereas hyperphosphorylation occurs at Ser12742 in the PEVK domain [30]. Alterations in the expression and phosphorylation patterns of titin isoforms significantly contribute to increased cardiomyocyte stiffness. Therefore, titin regulation is a major research focus. Key kinases modulating cardiomyocyte stiffness include alpha kinase 2, sGC, PKG, and PKA [30, 31, 32]. Genetic inhibition of RBM20 promotes N2BA expression and significantly reduces myocardial passive tension [33]. These findings suggest that titin-targeted gene editing technologies (e.g., CRISPR) and novel biomarkers, such as matrix metalloproteinase-12 (MMP-12)–cleaved titin fragments, should be further investigated [34]. The subtypes and phosphorylation of titin are the main factors determining myocardial stiffness in HFpEF, and the goal of drug therapy is to improve these parameters. Because myocardial biopsy is an invasive procedure, it is not recommended for patients with preserved ejection fraction heart failure who can be diagnosed clearly based on the patient’s condition, common laboratory tests, and imaging examinations. Therapeutic effects should be evaluated not only through biopsy, molecular profiling, and gene editing but also based on laboratory indicators and the symptoms and signs of disease.

In addition to titin, aberrant stabilization of the microtubule network can contribute to myocardial stiffness. Increased levels of detyrosinated α-tubulin increase microtubule rigidity, impeding cardiomyocyte relaxation [35]. On the contrary, inhibition of tubulin detyrosination reduces myocardial stiffness and accelerates relaxation [36], suggesting that restoration of microtubule dynamic equilibrium is a promising therapeutic strategy for HFpEF.

ECM, deposited between cardiomyocytes, provides structural support (Fig. 1). Aberrant accumulation of ECM components, particularly elastin and collagen, leads to myocardial interstitial fibrosis. This fibrosis reduces cardiac tissue compliance and is a prominent pathological feature observed in endomyocardial biopsies from 93% of patients with HFpEF [37]. Cardiac fibrosis is closely associated with neurohumoral regulation abnormalities, inflammation, metabolic dysregulation, and intracellular molecular pathways. Oxidative stress stimulates fibroblast activation and pathological ECM remodeling primarily through the activation of the fibrogenic transforming growth factor-beta (TGF-β) signaling pathway [38].

## 3. Advances in the Treatment of HFpEF

### 3.1 Guideline-guided Drug Treatment for HFpEF

Renin–angiotensin system (RAS) inhibitors are important treatment agents for HFpEF. However, the long-term outcomes of conventional RAS inhibitors in HFpEF have shown mixed results. For example, in the CHARM-Preserved trial, patients with chronic heart failure (CHF), New York Heart Association (NYHA) functional classes II–IV, and left ventricular ejection fraction (LVEF) of >40% were randomly assigned to candesartan and placebo groups. The proportion of patients with one or more hospitalizations for CHF was lower in the candesartan group than in the placebo group (230 vs 279, *p* = 0.017); however, no significant difference was observed in the number of cardiovascular deaths [39]. A meta-analysis including 12 studies with a total of 30,882 patients (16,540 in the RAS inhibitor group and 14,432 in the control group) showed that RAS inhibitor therapy was associated with a significantly reduced risk of the primary composite outcome (odds ratio [OR], 0.87; 95% confidence interval [CI], 0.82–0.93) and hospitalization for heart failure (OR, 0.84; 95% CI, 0.75–0.94). However, RAS inhibitor therapy had no significant effect on the risk of mortality (OR, 0.79; 95% CI, 0.55–1.12) [40].

Among RAS inhibitors, the therapeutic effects of angiotensin receptor–neprilysin inhibitors (ARNIs) on hypertension and heart failure have gradually received attention. Sacubitril–valsartan, a representative ARNI, can not only block angiotensin receptors but also inhibit neprilysin. Mechanistic studies have shown that sacubitril–valsartan enhances titin phosphorylation by activating the cgPM–PKG pathway and improves myocardial stiffness in diabetic mice [41]. In addition, it can improve diastolic function by reversing ventricular hypertrophy and reducing the overall strain on the heart in HFpEF [42, 43]. The PARAGON-HF trial showed that sacubitril–valsartan was more effective than valsartan alone in reducing N-terminal pro-B-type natriuretic peptide (NT-proBNP) levels in patients with HFpEF and significantly improved the NYHA class [44, 45]. A meta-analysis indicated that sacubitril–valsartan reduced the incidence of decompensated heart failure and composite decompensated heart failure/all-cause mortality but increased the risk of hypotension [46].

Whether the use of β-blockers for treating HFpEF can provide long-term benefits is controversial. A study from the Swedish Heart Failure Registry showed that β-blockers did not significantly reduce the risk of hospitalization for heart failure or cardiovascular death despite their use in up to 80% of patients with HFpEF [47]. A secondary analysis of the TOPCAT trial showed that β-blockers did not affect events in patients with heart failure and LVEF of 45%–49% but increased the risk of hospitalization in patients with heart failure and LVEF of ≥50% [48]. However, β-blockers are not entirely beneficial for patients with HFpEF. In a study on patients with heart failure with mildly reduced ejection fraction (HFmrEF) and HFpEF, Matsumoto *et al*. [49] found that β-blocker use reduced the risk of cardiovascular death or hospitalization for heart failure in patients with heart failure and atrial fibrillation. Although existing studies have not shown any long-term benefits of β-blockers in patients with HFpEF, these drugs may have potential value in specific subgroups of HFpEF (e.g., those with concomitant atrial fibrillation). The therapeutic efficacy of β-blockers in HFpEF may be highly dependent on patient characteristics; therefore, future studies should perform precise phenotypic analysis to identify patients eligible for β-blocker therapy.

Jan-Christian Reil *et al*. [50] found that increased insulin and blood glucose levels in diabetic mice (db/db) led to increased vascular stiffness, which was associated with increased heart rate, disturbed ventricular–arterial coupling, and diastolic dysfunction. At the pathological level, the N2B isoform of titin was significantly upregulated in the cardiomyocytes of db/db mice, which was one of the causes of cardiac diastolic dysfunction. When db/db mice were administered the If channel inhibitor ivabradine, the heart rate decreased, the ventricular–arterial coupling disorder was corrected, and the expression of N2B in cardiomyocytes was downregulated, resulting in the recovery of diastolic function. However, a meta-analysis on the use of ivabradine in human HFpEF failed to replicate these benefits [51]. This discrepancy in the therapeutic effects of ivabradine in animal HFpEF models and patients with HFpEF is related to species differences, comorbidities, and subgroup analysis. Although the mechanisms underlying cardiac and vascular lesions in diabetic mice overlap with those underlying HFpEF, they are different from those of human HFpEF. Although db/db mice showed reduced cardiac diastolic function, they cannot completely mimic the diastolic dysfunction observed in HFpEF. Therefore, these findings can be used only as a reference for further clinical research and not as an indication for the clinical application of ivabradine. In addition, in animal models, the use of ivabradine is based on a significantly elevated heart rate; however, in clinical settings, only a few patients with HFpEF have heart rates of >80. Therefore, future clinical studies should investigate the effects of drug therapy on patients with HFpEF with arrhythmia or abnormally high heart rates to clarify the indications for antiarrhythmic medications.

### 3.2 Mineralocorticoid Receptor Inhibitors

Overactivation of the mineralocorticoid receptor (MR) promotes myocardial fibrosis, arterial stiffening, and inflammatory responses. Its role in HFpEF has attracted considerable attention [52]. The FINEARTS-HF trial showed that finerenone reduced the risk of cardiovascular death and worsening heart failure in patients with LVEF values of ≥40%, with consistent efficacy across LVEF subgroups [53].

### 3.3 Aldosterone Receptor Antagonists

A post-hoc analysis of the TOPCAT trial indicated that spironolactone can improve clinical outcomes in patients with HFpEF with specific phenotypes, such as those exhibiting increased levels of inflammatory markers or myocardial fibrosis [54]. Kosmala *et al*. [55] analyzed the effects of spironolactone on exercise capacity in patients with HFpEF and found that 6 months of treatment improved left ventricular untwisting rate and the E/eʹ ratio. Moreover, the improvement in the E/eʹ ratio was independently correlated with increased peak VO_2_, suggesting that spironolactone functions by suppressing aldosterone-mediated myocardial remodeling. In addition, spironolactone use was associated with a 17% reduction in the hospitalization rate for heart failure [56].

### 3.4 Sodium–glucose Cotransporter 2 Inhibitors and Other Antidiabetic Drugs

Sodium–glucose cotransporter 2 (SGLT2) inhibitors are established as first-line drugs for HFpEF. The EMPEROR-Preserved trial showed that empagliflozin significantly reduced the risk of cardiovascular death or hospitalization for heart failure in patients with HFpEF [57]. The DELIVER trial indicated that dapagliflozin reduced the risk of worsening heart failure or cardiovascular death, with consistent efficacy in patients with LVEF values of ≥50% [58]. A meta-analysis involving patients with HFpEF and HFmrEF showed that SGLT2 inhibitors, ARNIs, and MRAs significantly reduced the risk of hospitalization for heart failure, with SGLT2 inhibitors exhibiting the strongest efficacy in achieving this outcome [59]. Mechanistic studies have indicated that empagliflozin significantly suppresses cardiomyocyte inflammation and ameliorates pathological oxidative alterations in both cytosol and mitochondria, consequently reversing titin hypophosphorylation and improving cardiomyocyte stiffness in HFpEF [60]. In addition, empagliflozin can alleviate cardiac hypertrophy by regulating autophagy, thereby improving cardiac diastolic function [61]. Maximilian Trum *et al*. [62] assessed sodium influx in cardiac biopsies from patients with HFpEF. Results indicated that patients with HFpEF had significantly increased late sodium current; however, treatment with empagliflozin reduced sodium influx and late sodium current. These findings suggest that empagliflozin has therapeutic potential for HFpEF and arrhythmia. Dapagliflozin exerts beneficial effects in HFpEF by activating AMP-activated protein kinase (AMPK) and inhibiting the mammalian target of rapamycin (mTOR) pathway, thereby suppressing NO-induced oxidative stress, pro-inflammatory cytokines, myocardial hypertrophy, and fibrosis [63].

In addition to SGLT2 inhibitors, glucagon-like peptide-1 (GLP-1) receptor agonists have shown promise in the treatment of HFpEF. The STEP-HFpEF trial showed that semaglutide significantly improved quality of life in obese patients with HFpEF, evidenced by an increased Kansas City Cardiomyopathy Questionnaire Clinical Summary Score (KCCQ-CSS) and improved 6-minute walk distance [64]. The SUMMIT trial indicated that the dual GIP/GLP-1 receptor agonist tirzepatide improved quality of life and reduced the risk of cardiovascular death or worsening heart failure in patients with HFpEF [65]. In addition, metformin has been shown to improve diastolic function in mice with transverse aortic constriction/deoxycorticosterone acetate (TAC/DOCA)–induced HFpEF by increasing N2B phosphorylation [66].

### 3.5 Potential Therapeutic Strategies for HFpEF

Angiotensin-converting enzyme 2 (ACE2), a homolog of ACE, is a monocarboxypeptidase that converts Ang II into Ang 1–7. ACE2 and Ang 1–7 negatively regulate RAS at two nodes. JiuChang Zhong *et al*. [67] infused mice with Ang II to induce HFpEF. They found that elevated Ang II levels induced hypertension, myocardial hypertrophy, fibrosis, and diastolic dysfunction, whereas ACE2 administration restored diastolic function by attenuating the pathological effects of excess Ang II. Furthermore, Ang 1–7 can ameliorate cardiac diastolic dysfunction by improving endothelial function, reducing myocardial fibrosis, and reversing cardiac hypertrophy in db/db mice [68]. These findings indicate that ACE2 and Ang 1–7 are potential treatments for HFpEF. The successful completion of phase I (NCT00886353) and II (NCT01597635) clinical trials of ACE2 has provided key translational evidence for the potential use of race2 as a therapeutic agent.

As mentioned earlier, inflammation is involved in the development of HFpEF. The D-HART study, inspired by the treatment of rheumatoid arthritis, investigated the therapeutic value of the IL-1 blocker anakinra in HFpEF [69]. Patients with HFpEF were randomly divided into anakinra (100 mg) and placebo groups. Results revealed that anakinra significantly reduced systemic inflammation and improved exercise capacity in patients with HFpEF with high levels of inflammatory markers. No major adverse events were observed, except for mild and self-limited injection site reactions in three patients. These findings suggest that anti-inflammatory drugs can improve the quality of life of patients with HFpEF with increased inflammatory marker levels to some extent. However, the sample size of the study was small, and the follow-up duration was 1 month. Moreover, the long-term effects of anakinra on HFpEF remain unclear. Therefore, the depth and breadth of anakinra-related clinical research should be expanded based on these findings. β-hydroxybutyrate can alleviate myocardial fibrosis and improve diastolic function in mice with HFpEF by inhibiting NOD-, LRR- and pyrin domain-containing protein 3 (NLRP3) inflammasome and restoring mitochondrial acetylation balance [70]. The dipeptidyl peptidase 4 (DPP4) inhibitor sitagliptin can improve diastolic function by inhibiting inflammatory signaling pathways and reducing endothelial oxidative stress in Dahl salt-sensitive rats fed a high-salt diet [71]. However, these studies remain limited to animal experiments. Future studies should investigate the therapeutic effects and safety of anti-inflammatory drugs such as β-hydroxybutyrate and sitagliptin in HFpEF in clinical settings.

Miyamoto *et al*. [72] found that TY1, a synthetic non-coding RNA drug, improved cardiac diastolic function in mice with HFpEF through sustained inhibition of oxidative stress–induced mitogen-activated protein kinase (MAPK) signaling and expression of downstream inflammatory, fibrosis-related, and hypertrophy-related genes in cardiac tissue, with oral and intravenous administration showing comparable effects. Although non-coding RNA therapy is promising, existing studies are limited to animal experiments. Whether it has the same therapeutic effects on human HFpEF remains unclear; therefore, its clinical applicability warrants investigation.

Cardiac systolic and diastolic function are intricately related to energy metabolism. Trimetazidine inhibits long-chain 3-ketoacyl coenzyme A thiolase to shift cardiac energy metabolism from fatty acid oxidation to glucose oxidation, which is more energy efficient and is theoretically favorable for both contraction and relaxation. However, the DoPING-HFpEF study showed that trimetazidine did not improve myocardial energy homeostasis or exercise hemodynamics in patients with HFpEF [73]. Theoretically, phosphodiesterase type 5 inhibitors (PDE5is), such as sildenafil, increase intracellular cGMP concentrations, thereby protecting endothelial function. Animal studies have shown that sildenafil suppresses left ventricular remodeling, hypertrophy, and fibrosis. However, the RELAX trial did not show clinical benefits of phosphodiesterase type 5 (PDE5) inhibition in patients with HFpEF [74]. The mechanisms underlying the development of HFpEF are very complex and not limited to energy metabolism, which may be the reason for the negative results of the DoPING HFpEF and RELAX studies. On the contrary, clinical trials of sGC stimulants have shown more promising results. The DYNAMIC trial showed that the sGC stimulator riociguat improved hemodynamic characteristics but had limited efficacy in alleviating symptoms in patients with HFpEF with pulmonary hypertension [75]. The SOCRATES-PRESERVED trial showed that the sGC stimulator vericiguat improved KCCQ-CSS scores in a dose-dependent manner [76].

Intravenous administration of ferric carboxymaltose (FCM) can reduce the levels of oxidative stress markers (e.g., malondialdehyde) and improve endothelial function [77]. The FAIR-HFpEF trial showed that FCM increased the 6-minute walk distance and reduced the incidence of serious adverse events [78]. A retrospective study showed that FCM improved LVEF and increased right ventricular function normalization rates in patients with HFpEF [79]. Larger-scale trials are warranted to validate the long-term benefits of iron supplementation in HFpEF. Based on the findings of existing studies, FCM should be used in patients with HFpEF after assessing serum iron levels.

In the PIROUETTE trial, oral administration of the antifibrotic drug pirfenidone significantly reduced myocardial extracellular volume in patients with HFpEF [80]. In addition to pirfenidone, statins can inhibit fibrosis and inflammation. For instance, simvastatin can suppress the phosphorylation of Smad2/3 and MAPK pathways downstream of TGF-β signaling, thereby reducing collagen deposition, alleviating fibrosis, and improving diastolic function in mice with HFpEF [81]. A study on hypertensive rats with left ventricular hypertrophy showed that rosuvastatin improved cardiac compliance by reducing interstitial fibrosis, suggesting that statins are more effective during early-stage HFpEF (before severe myocardial remodeling) than during established hypertrophy in later stages [82]. Furthermore, patients with HFpEF receiving simvastatin for 6 months show significant reductions in CRP and IL-6 levels, with larger reductions observed in patients exhibiting severely impaired diastolic function (pseudonormalization) [83]. These findings indicate that statins exert therapeutic effects against HFpEF through anti-inflammatory mechanisms.

### 3.6 Non-pharmacological Interventions

Numerous recent studies have shown that exercise training is a crucial non-pharmacological intervention for improving cardiac diastolic function and exercise capacity in patients with HFpEF [84]. Roeder *et al*. [85] found significantly reduced left atrial conduit strain in patients with HFpEF, which was strongly correlated with peak VO_2_. High-intensity exercise training (HIIT) significantly reduces left ventricular myocardial stiffness and increases peak oxygen uptake [86]. Both HIIT (comprising a warm-up of 10 min at moderate intensity, four intervals of 4 min at high intensity, alternating with three intervals, and a 3-min cool-down phase at moderate intensity, totaling 38 min) and moderate-intensity continuous training (moderate-intensity exercise for 47 minutes) can improve the E/eʹ ratio and quality of life, with HIIT leading to greater improvement in VO_2_ [87]. HIIT rapidly enhances exercise capacity by upregulating the activity of enzymes involved in skeletal muscle energy metabolism and enhancing satellite cell function [88]. Furthermore, low-intensity training improves exercise tolerance in pigs with HFpEF by inhibiting MMP-2 and increasing type III collagen expression, thereby alleviating myocardial fibrosis and enhancing diastolic function [89]. The aforementioned clinical studies on exercise training were performed under the guidance of professional coaches and clinicians. When their condition is stable, each patient with HFpEF should be prescribed a step-by-step exercise program under the guidance of professionals, and this program should be adjusted according to the changes in their condition and the degree of adaptation.

With the advancement of interventional techniques, studies have focused on neuromodulation for treating HFpEF. A study showed that renal denervation (RDN) decreased left ventricular diastolic stiffness, left ventricular filling pressure, and NT-proBNP levels at the 6-month follow-up in patients with HFpEF, indicating a significant effect of RDN on HFpEF [90]. The RDT-PEF trial showed that compared with control individuals, patients with HFpEF undergoing RDN showed greater improvements in peak VO_2_ and E/eʹ ratio after 3 months [91]. In a study on rats with obesity-induced cardiac dysfunction, early radiofrequency renal denervation (RF-RDN) (at 8 weeks) significantly reduced renal norepinephrine levels, delayed myocardial fibrosis, improved endothelial function, and ameliorated cardiac dysfunction. However, RF-RDN failed to exert beneficial effects when administered to 20-week-old rats with HFpEF [92]. These findings suggest that RDN should be initiated as soon as possible to achieve the best therapeutic effect against HFpEF.

In the REBALANCE-HF trial, splenic artery vasomodulation (SAVM) was performed on 18 patients with HFpEF. At 1 month, SAVM significantly reduced pulmonary capillary wedge pressure (PCWP) during exercise and improved KCCQ-CSS scores [93]. At 12 months, the number of hospitalizations for heart failure, motor function, and health status showed no significant differences between the SAVM and control groups, indicating that SAVM is safe and feasible for the treatment of HFpEF [94].

In another study, thoracoscopic ablation of the right greater splanchnic nerve was performed on 10 patients with heart failure with ejection fraction (EF) values of >40%. This intervention reduced PCWP during exercise by 4.5 mmHg at 3 months and significantly improved Minnesota Living with Heart Failure Questionnaire scores at 12 months [95]. However, three patients experienced procedure-related adverse events, highlighting the need for careful risk–benefit assessment. Furthermore, low-intensity transcutaneous vagus nerve stimulation (tVNS) can significantly improve global longitudinal strain, inflammatory cytokines, and quality of life in patients with HFpEF [96]. The ANTHEM-HF study showed that cervical vagus nerve stimulation improved NYHA class, 6-minute walk distance, and quality of life in patients with HFpEF and HFmrEF after 12 months, with a lower incidence of adverse events [97]. These findings suggest that vagus nerve stimulation can alleviate symptoms and improve the quality of life in patients with HFpEF.

The REDUCE LAP-HF II trial evaluated the therapeutic efficacy of an atrial shunt device in patients with HFpEF and HFmrEF. The shunt group showed a 5.65-mL reduction in left ventricular end-diastolic volume, an increase in right ventricular volume, and a reversal of ventricular remodeling without the worsening of right ventricular systolic function within 24 months [98]. These results suggest that atrial shunt therapy leads to more favorable changes in cardiac structure/function in patients with HFpEF. Furthermore, pericardiectomy can relieve pericardial constraint on left ventricular filling. In a porcine HFpEF model, pericardiectomy decreased the increase in left ventricular end-diastolic pressure from 13 ± 5 mmHg to 4 ± 3 mmHg, with a larger increase in left ventricular volume [99]. These findings provide novel insights for using minimally invasive therapy in the treatment of HFpEF. However, human safety data remain limited, and long-term efficacy, particularly the impact of pericardiectomy on pericardial regeneration, requires further investigation.

## 4. Limitations and Prospects

HFpEF management is primarily based on guideline-directed medical therapy (GDMT). Existing studies have revealed the complex mechanisms underlying HFpEF, particularly the interplay between inflammation, metabolic dysregulation, and cell death pathways, prompting the evaluation of novel treatments such as RNA-based therapies, kinase modulators, and interventional procedures. However, clinical trials remain limited by small sample sizes, high heterogeneity, or suboptimal endpoint designs without achieving translational breakthroughs. In addition, owing to the numerous complications of HFpEF, subgroup analysis of patients with HFpEF should be actively performed, and targeted treatment should be administered based on the results of the subgroup analysis. For instance, subgroup analysis should assess whether antiarrhythmic drugs can offer long-term benefits for patients with abnormally elevated heart rates.

Future studies should prioritize integrating multimodal therapeutic strategies, such as exercise training combined with targeted pharmacotherapy, to enhance synergistic therapeutic efficacy. Combination therapy should be administered after thoroughly examining patients and understanding the underlying etiological factors and complications. For example, SGLT2 inhibitors combined with exercise training can not only control blood glucose levels but also improve the quality of life and reduce the incidence of long-term adverse events in patients with HFpEF with diabetes. However, combination therapy should not be initiated hastily based on theoretical evidence. Further preclinical and clinical studies are warranted to evaluate the clinical applicability, safety, and effectiveness of combination therapy in patients with HFpEF.

## 5. Conclusion

The pathogenesis of HFpEF is intricately related to inflammatory activation, oxidative stress, and metabolic dysregulation. In addition to directly damaging vascular and myocardial cells, these mechanisms increase myocardial stiffness and aggravate cardiac interstitial fibrosis. This cascade eventually reduces cardiac compliance and impairs diastolic function.

Although clinical evidence supporting the use of GDMT for HFpEF remains limited, existing management strategies are primarily based on the established treatment regimens for HFrEF. Concurrently, novel therapeutic agents and strategies targeting the underlying pathophysiological mechanisms of HFpEF are being investigated. Although these studies are predominantly at the preclinical stage, they provide substantial support for the development of novel drugs for HFpEF.
